# Silymarin Prevents Restraint Stress-Induced Acute Liver Injury by Ameliorating Oxidative Stress and Reducing Inflammatory Response

**DOI:** 10.3390/molecules21040443

**Published:** 2016-04-01

**Authors:** Sou Hyun Kim, Dal-Seok Oh, Ji Youn Oh, Tae Gen Son, Dong Yeon Yuk, Young-Suk Jung

**Affiliations:** 1 College of Pharmacy, Pusan National University, Busan 609-735, Korea; hyunie9808@naver.com (S.H.K.); pooh7282@naver.com (J.Y.O.); 2 The K-herb Research Center, Korea Institute of Oriental Medicine, Daejeon 305-811, Korea; dalsoh@gmail.com; 3 Division for Research Center, Dongnam Institute of Radiological and Medical Science, Busan 619-953, Korea; tgson@hanmail.net; 4 College of Pharmacy, Chungbuk National University, Cheongju 361-763, Korea; julycosmos@gmail.com

**Keywords:** silymarin, restraint stress, acute liver injury, oxidative stress, inflammation

## Abstract

Silymarin is a flavonoid extracted from the milk thistle *Silybum marianum*. It has been reported to prevent liver injuries induced by various chemicals or toxins. Our recent study suggested that silymarin induces hepatic synthesis of glutathione by increasing cysteine availability, which may consequently contribute to increased antioxidant capacity of the liver. In the present study, we investigated the effects of silymarin on acute liver injury induced by restraint stress. Silymarin (100 mg/kg) was orally administered to BALB/c mice every 12 h (3 times in total). After the last dose, mice were subjected to restraint stress for 6 h, and serum levels of aspartate and alanine aminotransferases, and hepatic levels of lipid peroxidation were determined. Hepatic levels of sulfur-containing metabolites such as methionine, *S*-adenosylmethionine, cysteine, and glutathione were also measured. The level of pro-inflammatory mediators in both liver and serum was determined. To study the mechanism of the effects of silymarin, we assessed Jun *N*-terminal kinase (JNK) activation and apoptotic signaling. Restraint stress induced severe oxidative stress and increased mRNA levels of pro-inflammatory mediators; both effects of restraint stress were significantly inhibited by silymarin. Moreover, administration of silymarin significantly prevented acute liver injury induced by restraint stress by blocking JNK activation and subsequently apoptotic signaling. In conclusion, these results suggest that the inhibition of restraint stress–induced liver injury by silymarin is due at least in part to its anti-oxidant activity and its ability to suppress the inflammatory response.

## 1. Introduction

Stress can disrupt physiological homeostasis and induce a number of psychological and physical disorders [1,2,3]. In rodents, restraint stress increases susceptibility to xenobiotics [4,5], inhibits lipid and glucose metabolism [6,7], and provokes liver damage [8,9]. Excessive generation of reactive oxygen species (ROS) is the main contributor to stress-initiated diseases [9,10]. The liver is a major organ prone to oxidative stress-induced damage. On the basis of clinical observations, a correlation between hepatic disease and psychological stress has been proposed [11]. The authors have suggested that emotional stress worsens the symptoms of hepatic disorders and alters blood chemistry related to liver function [11]. In accordance with this suggestion, experimental models showed that psychological stress induces severe oxidative stress followed by liver damage [12,13].

Milk thistle (*Silybum marianum*; Asteraceae) has been used as a traditional medicine since ancient times to treat hepatic disorders, including hepatitis and cirrhosis, and to protect the liver against poisoning by chemicals and environmental toxins [14]. Silymarin, which is extracted from the seed of milk thistle, is a mixture of silybin, isosilybin, silydianin, and silychristin. It has been suggested that silymarin possesses a variety of pharmacological activities including anti-inflammatory, antifibrotic, and antioxidant activities. Indeed, experimental studies have proved the hepatoprotective effect of silymarin against toxic chemicals such as ethanol, diethylnitrosamine, and carbon tetrachloride [15,16,17]. These chemicals induce liver damage by inducing excessive generation of reactive metabolites, thus, the hepatoprotective effects of silymarin are frequently attributed to its antioxidant activity. Previous studies have shown that silymarin treatment ameliorates toxicant-induced glutathione (GSH) depletion in various models of experimental liver injury [15,16,17]. The effect of silymarin is usually thought to be indirect, resulting from the preservation of GSH, (which plays an important role in antioxidant defense), because silymarin is able to directly scavenge radicals. Our recent study has elucidated that silymarin regulates the metabolism of sulfur-containing amino acids to maintain the GSH pool through increased cysteine availability in the liver, which could be a potential mechanism of its antioxidant action in addition to radical scavenging [18]. In this study, we hypothesized that silymarin could prevent restraint stress-induced acute liver injury by activating the defense against oxidative stress-induced apoptosis.

## 2. Results

### 2.1. Effect of Silymarin on Restraint Stress-Induced Acute Liver Injury

**Figure 1:**
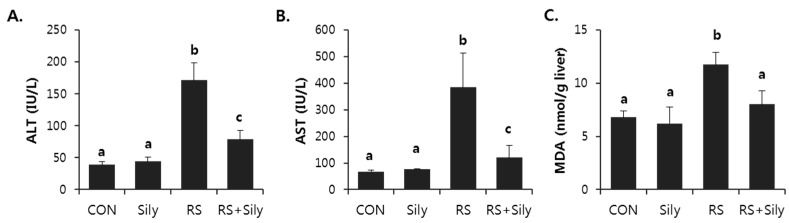
Silymarin inhibited restraint-induced acute liver injury. Serum activity of ALT (**A**) and AST (**B**); and hepatic level of MDA (**C**) were analyzed to examine liver injury. Each value is the mean ± SD for 4 mice. Values with different letters (a, b, c) are significantly different from one another (one-way ANOVA followed by Newman-Keuls multiple range test, *p* < 0.05). CON; control mice, Sily; only silymarin-treated mice, RS; restraint stressed mice, RS + Sily; restraint-stressed mice with silymarin treatment.

Serum alanine aminotransferase (ALT) and aspartate aminotransferase (AST) activities in the restraint-challenged mice were significantly higher than those in the control mice (Figure 1). Silymarin supplementation markedly reduced the increase in serum enzyme activities induced by restraint stress, indicating that silymarin administration protected against restraint stress-induced acute liver injury.

### 2.2. Effect of Silymarin on the Levels of Malondialdehyde (MDA) and 4-Hydroxynonenal (4-HNE) in the Liver of Stressed Mice

**Figure 2:**
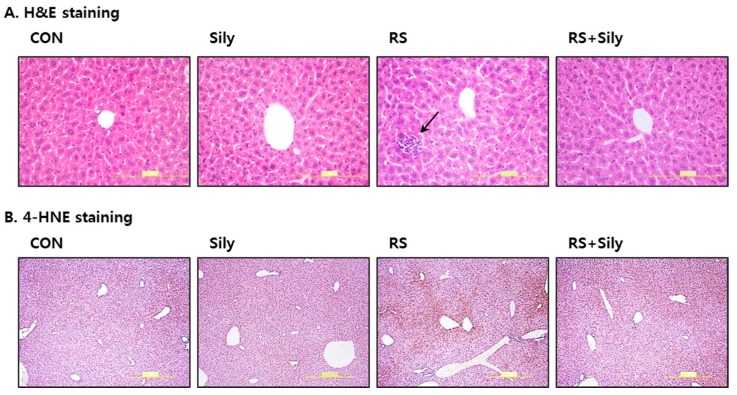
Effect of silymarin on histological changes of the liver in mice subjected to restraint stress. (**A**) Hematoxylin and eosin (H & E) staining of representative liver sections shown at the same magnification (400×). Only mice subjected to restraint showed infilterated cells in the liver parenchyma. Black arrow represents inflammatory cell infiltration; (**B**) Representative pictures of 4-HNE protein adducts with brown color. 4-HNE positivity was observed mostly in hepatocytes around the central vein and it was most clear in the liver of restraint stressed mice. (CON; control mice, Sily; only silymarin-treated mice, RS; restraint stressed mice, RS + Sily; restraint-stressed mice with silymarin treatment).

Several end-products of lipid peroxidation including MDA and 4-HNE have been used as biomarkers of oxidative stress. Restraint stress significantly increased MDA levels in the liver (Figure 1C) and increased the intensity of 4-HNE staining (Figure 2); both effects were prevented by treating stressed mice with of silymarin.

### 2.3. Effect of Silymarin on Restraint-Induced GSH Depletion

**Figure 3:**
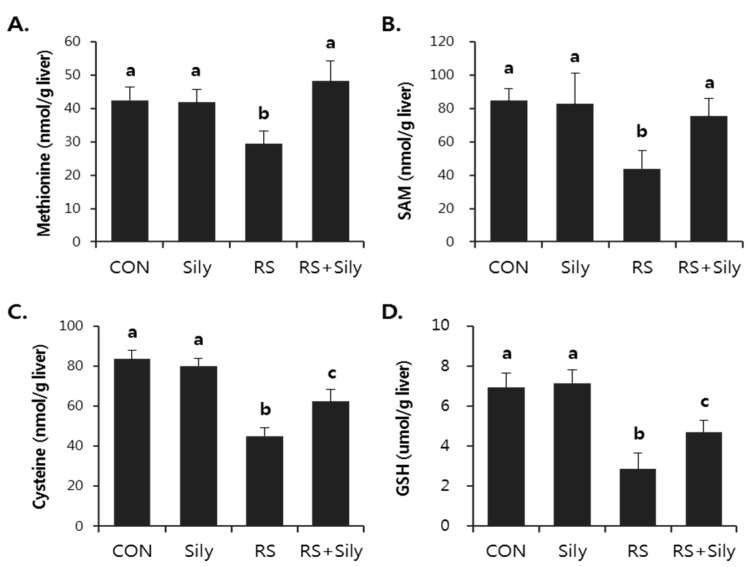
Silymarin ameliorated restraint-induced GSH depletion via down-regulation of sulfur-containing amino acid metabolism. Hepatic level of methionine (**A**); SAM (**B**); cysteine (**C**); and GSH (**D**) were determined. Each value is the mean ± SD for four mice. Values with different letters (a, b, c) are significantly different from one another (one-way ANOVA followed by Newman-Keuls multiple range test, *p* < 0.05). CON; control mice, Sily; only silymarin-treated mice, RS; restraint stressed mice, RS + Sily; restraint-stressed mice with silymarin treatment.

Restraint stress significantly suppressed hepatic GSH levels (Figure 3D). However, the pretreatment of stressed mice with silymarin prevented the depletion of GSH and was accompanied by the recovery of the levels of upstream metabolites such as methionine (Figure 3A), S-adenosylmethionine (SAM) (Figure 3B), and cysteine (Figure 3C).

### 2.4. Effect of Silymarin on the Expression of Inflammatory Mediator Genes in the Liver of Stressed Mice

**Figure 4:**
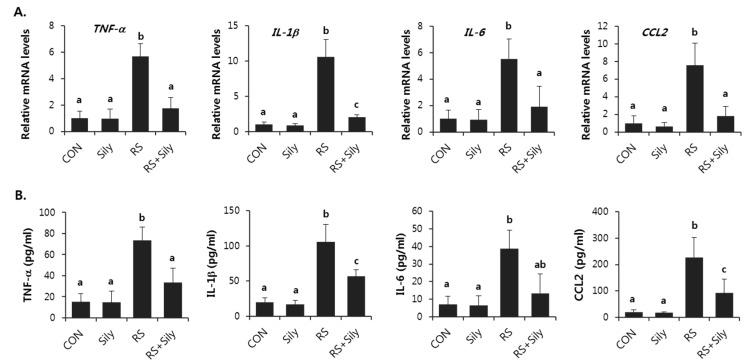
Silymarin prevented increase in mRNA (**A**) and serum levels (**B**) of pro-inflammatory mediators induced by restraint stress. Each value is the mean ± SD for four mice. Values with different letters (a, b, c) are significantly different from one another (one-way ANOVA followed by Newman-Keuls multiple range test, *p* < 0.05). CON; control mice, Sily; only silymarin-treated mice, RS; restraint stressed mice, RS + Sily; restraint-stressed mice with silymarin treatment.

The expression of TNF-α, IL-1β, IL-6, and CCL2 mRNAs was significantly increased by restraint stress in mice, whereas silymarin treatment clearly reversed the restraint stress-induced increase in the expression of these genes in the liver (Figure 4A). In accordance with this, silymarin significantly prevented increased serum levels of TNF-α, IL-1β, IL-6, and CCL2 induced by restraint stress (Figure 4B).

### 2.5. Effect of Silymarin on JNK Activation and Apoptotic Pathway in the Liver of Stressed Mice

**Figure 5:**
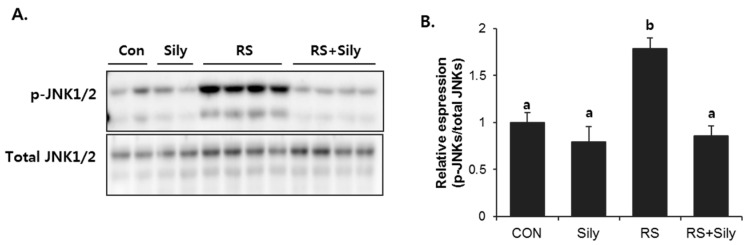
Silymarin treatment prevented restraint-induced JNKs phosphorylation. (**A**) Immunoblot analysis of phopho-JNK1/2 and total JNK1/2. (**B**) Quantitative analysis of blots. Each value is the mean ± SD for four mice. Values with different letters (a, b) are significantly different from one another (one-way ANOVA followed by Newman-Keuls multiple range test, *p* < 0.05). CON; control mice, Sily; only silymarin-treated mice, RS; restraint stressed mice, RS + Sily; restraint-stressed mice with silymarin treatment.

**Figure 6:**
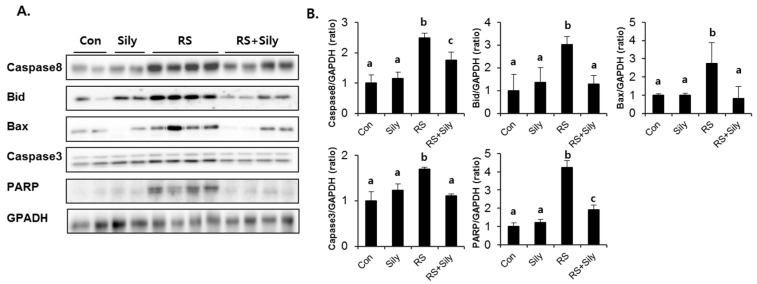
Silymarin treatment reduced restraint-induced apoptotic cascade. (**A**) Immunoblot analysis of caspase8, Bid, Bax, caspase3, PARP, and GAPDH. (**B**) Quantitative analysis of blots. Each value is the mean ± SD for four mice. Values with different letters (a, b) are significantly different from one another (one-way ANOVA followed by Newman-Keuls multiple range test, *p* < 0.05). CON; control mice, Sily; only silymarin-treated mice, RS; restraint stressed mice, RS + Sily; restraint-stressed mice with silymarin treatment.

Activation of Jun N-terminal kinases (JNKs) is known to induce apoptosis. To investigate whether inhibition of JNK signaling was involved in the effect of silymarin, we determined the effects of silymarin on JNK activation in the liver of stressed mice, which was estimated as the level of JNK phosphorylation. Restraint stress dramatically increased phosphorylation of JNKs, whereas silymarin significantly downregulated their phosphorylation to the control levels (Figure 5). As we expected, JNK phosphorylation was correlated with the activation of the apoptotic cascade (Figure 6). The expression of caspase 8, Bid, Bax, and caspase 3, and PARP cleavage were all induced by restraint stress, while silymarin treatment efficiently blocked these effects.

## 3. Discussion

Restraint stress is known to induce oxidative stress followed by liver injury. A number of studies have focused on the pathogenic role of oxidative stress in restraint stress-induced liver injury and on the preventive and/or therapeutic intervention by using antioxidants. Silymarin has been used as traditional medicine for a long time and it was uncovered that its protective effect against liver injury appears to rely on defense against oxidative stress and inflammatory response. In particular, our recent study elucidated that silymarin enhanced GSH synthesis by increasing the availability of cysteine, the essential substrate for GSH generation. Considering that GSH is a potent endogenous antioxidant, we hypothesized that silymarin could prevent restraint stress–induced acute liver injury by reducing oxidative stress.

In this study, restraint stress in mice dramatically depleted hepatic GSH, whereas silymarin treatment significantly recovered restraint-induced GSH depletion presumably by increasing the levels of methionine, SAM, and cysteine, which are upstream metabolites that are needed to synthesize GSH. In addition, silymarin prevented restraint-induced oxidative stress, as evidenced by the decreased levels of MDA and 4-HNE in the liver. We therefore suggest that the hepatoprotective effects of silymarin might be associated with augmentation of antioxidant defense against oxidative stress induced by restraint stress via the improvement of impaired metabolism of sulfur-containing amino acids.

Oxidative stress can up-regulate pro-inflammatory mediators and trigger an inflammatory response, which aggravates liver injury [19]. In the early phase after a hazardous incident that generates excessive ROS, hepatic macrophages rapidly secrete pro-inflammatory cytokines and chemokines such as TNF-α, IL-1β, IL-6, and CCL2, resulting in apoptotic signaling [20]. TNF-α is involved in cellular injury and is thought to be a critical factor of inflammatory damage in liver diseases including viral hepatitis, alcoholic liver disease, nonalcoholic fatty liver disease, and ischemia-reperfusion injury [21,22]. IL-1β is a member of the IL-1 family that is best studied in the pathogenesis of liver injury. In experimental models, IL-1β promoted liver steatosis and fibrosis [23,24]. IL-6 is a pro-inflammatory cytokine produced by hepatocytes, adipocytes, and immune and endothelial cells [25,26]. Psychological stress can elevate the concentrations of IL-6 [27,28]. CCL2 (chemokine ligand 2) recruits and activates monocytes and macrophages to the site of tissue injury and regulates the pro-inflammatory cytokines TNF-α, IL-1β, and IL-6 [29,30]. Increased expression of CCL2 in the liver has been shown in acute and chronic inflammatory diseases and in numerous animal models of injury. Generally, CCL2 appears to play a critical role in hepatic inflammatory responses during liver injury [31,32]. In the present study, silymarin inhibited the expression of these pro-inflammatory mediators induced by restraint stress. Therefore, we suggest that silymarin has anti-inflammatory ability, which also contributed to its ability to attenuate liver damage induced by restraint stress.

Of interest, restraint stress significantly activated JNKs and was accompanied by the induction of apoptotic signaling; these effects were efficiently blocked by silymarin treatment. JNKs, which belong to the superfamily of MAP-kinases, play a critical role in death receptor-initiated extrinsic as well as mitochondrial intrinsic apoptotic pathways [33]. JNKs activate apoptotic cascade by upregulating the expression of pro-apoptotic genes or regulating the mitochondrial pro- and anti-apoptotic proteins by differential phosphorylation. Numerous studies have shown that both oxidative stress and pro-inflammatory cytokines can induce apoptosis by activating the JNK signaling pathway [33,34]. In line with these reports, we suggest that silymarin can prevent restraint stress–induced apoptotic signaling by inhibiting JNK activation.

## 4. Materials and Methods

### 4.1. Animals and Treatments

Male BALB/c mice, weighing 22–27 g, were purchased from Orient-Bio (Sungnam, Korea). The use of these animals was in compliance with the guidelines established by the Animal Care Committee in College of Pharmacy, Pusan National University, and approved by the Ethical Animal Care and Use Committee of Pusan National University (No. PNU-2015-0837). The mice were acclimated to temperature (22 ± 2 °C) and humidity (55% ± 5%) controlled rooms with a 12-h light/dark cycle for 1 week before use. Mice were gavaged with silymarin (100 mg/kg) every 12 h for a total of 3 doses. Silymarin was obtained from Sigma Aldrich (St. Louis, MO, USA) and prepared in a solvent composed of 10% propylene glycol, 12% Tween-80, and 78% distilled water. Control and restraint stressed mice received the vehicle only. After the last dosing, the mice were placed in Plexiglas restraint cages to induce restraint stress. The restraint cage was well ventilated and prevented animals from turning or ambulating, but it did not squeeze the mice. At the beginning of the restraint period, food and water were removed from control and restrained animals. After 6 h of restraint, all mice were sacrificed for collection of blood and liver samples.

### 4.2. Determination of Hepatotoxicity and Lipid Peroxidation

Serum activities of ALT and AST were determined by the method of Reitman and Frankel [35]. Malondialdehyde (MDA) levels were measured using a HPLC method [36]. Liver was homogenized in a threefold volume of cold 1.15% KCl. An aliquot of lysate was mixed with 0.2% thiobarbituric acid in 2 M sodium acetate buffer, pH 3.5, containing 5% butylated hydroxytoluene in ethanol. The mixtures were incubated at 95 °C for 45 min. After centrifugation, the supernatant was injected into HPLC equipped with a fluorescence detector (FLD-3100; Thermo Scientific, Sunnyvale, CA, USA) and a 5 μm Symmetry C_18_ reversed phase column (4.6 mm × 150 mm; Eka Chemicals, Bohus, Sweden). The mobile phase was composed of 35% methanol and 65% 50 mM sodium phosphate buffer, pH 7.0. The MDA–thiobarbituric acid complex was monitored by fluorescence detection with excitation at 515 nm and emission at 553 nm.

### 4.3. Liver Histopathology

For histopathologic analysis, small pieces of liver were fixed in 10% phosphate-buffered formalin. The fixed tissues were subsequently processed with an automatic tissue processor and embedded in paraffin blocks. 10 µm of tissue sections were stained with hematoxylin-eosin (HE) and 4-HNE, followed by microscopic examination.

### 4.4. Determination of Sulfur-Containing Amino Acids

The liver was homogenized in 1 M HClO_4_ for detection of SAM, cysteine, and GSH, or in methanol for detection of methionine. After the denatured protein was removed by centrifugation at 10,000× *g* for 10 min, the supernatant was assayed for the total GSH concentration using a HPLC separation/fluorometric detection method [37]. Cysteine was determined by the acid-ninhydrin method [38]. An incubation mixture consisting of acid-ninhydrin solution, acetic acid, and liver sample was incubated at 100 °C for 10min. After cooling, 95% ethanol (*v*/*v*) was added followed by the absorbance measurement at 560 nm. Methionine was derivatized with ophthaldialdehyde/2-mercaptoethanol prior to quantification using the method of Rajendra [39]. An HPLC system installed with a 3.5-μm Kromasil C_18_ column (4.6 × 100 mm; Eka Chemicals), and a fluorescence detector (FLD-3100; Thermo Scientific) was used. The method of She *et al.* [40] was employed to quantify SAM. A liver sample was injected into an HPLC system with a 3.5-μm Kromasil C_18_ column (4.6 × 250 mm; Eka Chemicals) and analyzed by a UV detector (UV-3000; Thermo Scientific).

### 4.5. Measurement of Cytokines in Serum

The levels of serum TNF-α, IL-1β, IL-6, and CCL2 were quantitated by enzyme-linked immunosorbent assay using a commercially available kit (R & D Systems, Minneapolis, MN, USA). Each serum was assayed in duplicate according to the manufacturer’s instruction.

### 4.6. RNA Extraction and Real-Time RT-PCR

Total RNA was purified from liver tissue using the RNeasy kit (Qiagen, Valencia, CA, USA). cDNA synthesis was accomplished with iScript™ cDNA Synthesis system (Bio-Rad, Hercules, CA, USA). Thunderbird SYBR qPCR mix (Toyobo Co., Ltd., Osaka, Japan) was used for performing Real time RT-PCR according to the manufacturer’s protocol. Relative values of gene expression were normalized to 18S ribosomal RNA. Primer sequences and full name of the genes are provided in Table 1.

### 4.7. Immunoblot Analysis

Liver tissues were lysed with ice-cold PRO-PREP™ protein extract solution (iNtRON, Sungnam, Gyunggi, Korea) and protein concentration was quantified using the BCA procedure (Thermo scientific). Equal amounts of protein samples were separated by SDS-PAGE and then transferred onto a polyvinylidene difuoride (PVDF) membrane (Millipore, Billerica, MA, USA). The membrane was blocked with 5% skim milk in 100 mM Tris-HCl (pH 7.5), 150 mM NaCl, and 0.2% Tween-20 (TBST) for 1 h at room temperature. The membranes were incubated with TBST containing 5% milk and the primary antibodies. After three washes with TBST, the blot was incubated with the appropriate horseradish peroxidase-conjugated secondary antibodies. The antigen was detected using an Western Bright ECL HRP substrate kit (Advansta, Menlo Park, CA, USA).

### 4.8. Data Analysis

All results expressed as the mean ± SD were analyzed by an oneway ANOVA followed by the Newman-Keuls multiple range test. The acceptable level of significance was established at *p* < 0.05.

## 5. Conclusions

The present study showed that oral administration of silymarin could prevent restraint stress-induced liver injury, and the mechanism is associated with its anti-oxidant action and anti-inflammatory activities. Further studies are needed to elucidate the pharmacologic effects of silymarin on other tissue damage by stress.

## Figures and Tables

**Table 1:** List of mouse primer used for real-time PCR.

Genes	Primer Sequences	
*TNF-α*	F: GGCCTCTCTACCTTGTTGCC	R: CAGCCTGGTCACCAAATCAG
*IL-1β*	F: TTCACCATGGAATCCGTGTC	R: GTCTTGGCCGAGGACTAAGG
*IL-6*	F: TTGCCTTCTTGGGACTGATG	R: CCACGATTTCCCAGAGAACA
*CCl2*	F: CCAGCAAGATGATCCCAATG	R: CTTCTTGGGGTCAGCACAGA
*18S*	F: CAGCCACCCGAGATTGAGCA	R: TAGTAGCGACGGGCGGTGTG
